# Rapid survey to determine the predictive factors of vaccination coverage in children aged 0 to 59 months in Guinea

**DOI:** 10.4102/sajid.v36i1.261

**Published:** 2021-08-26

**Authors:** Abdoulaye Touré, Ibrahima Camara, Alioune Camara, Mariama Sylla, Mamadou S. Sow, Alpha K. Keita

**Affiliations:** 1 Chaire de Sante Publique, Département des Sciences Pharmaceutiques, Université Gamal Abdel Nasser, Conakry, Guinea; 2 Centre de Recherche et de Formation en Infectiologie de Guinée (CERFIG), Université Gamal Abdel Nasser, Conakry, Guinea; 3 Institut National de Santé Publique (INSP), Ministère de la Santé, Conakry, Guinea; 4 Chaire de Sante Publique, Département de Sciences Médicales, Université Gamal Abdel Nasser, Conakry, Guinea; 5 Service de Maladies infectieuses et Tropicales, Hôpital National Donka, Conakry, Guinea; 6 TransVIHMI, Université de Montpellier/IRD/INSERM, Montpellier, France

**Keywords:** survey, vaccination, children, pre-COVID-19, Guinea

## Abstract

**Background:**

The Expanded Program on Immunisation has made it possible to prevent more than 3 million deaths in children under 5 years. The objectives of this study were to estimate the vaccination coverage of children from 0 to 59 months and identify factors associated with incomplete vaccination coverage.

**Methods:**

A cross-sectional study was carried out in a dispensary in Conakry, Guinea between January and February 2020. Sociodemographic and vaccination information was collected from mothers of 380 randomly select children aged 0 to 59 months. Information on immunisation coverage was gathered from records vaccination cards and maternal reports. Logistic regression was used to identify factors independently associated with incomplete immunisation coverage.

**Results:**

Most (66.5%) children aged < 12 months were up-to-date with their vaccinations. Factors associated with incomplete vaccination in this age group included: unavailability of vaccination cards (adjusted odds ratio [aOR] 7.58; 95% confidence interval [CI]: 2.56–22.44) and lack of prenatal consultation attendance (aOR 2.93; 95% CI: 1.15–7.48). In contrast only 19.8% (95% CI: 13.9–26.7) of children aged 12–59 months were fully immunised. Factors associated with incomplete vaccination coverage in children aged 12–59 months included high birth order (aOR 10.23; 95% CI: 2.06–19.43), and lack of prenatal consultation attendance (aOR 5.34; 95% CI: 1.48–19.23).

**Conclusion:**

Child immunisation coverage is low in Guinea. These results highlight the need to develop strategies based on an integrated approach to overcome obstacles to childhood immunisation in Guinea.

## Introduction

Vaccination is recognised as one of the most effective measures to prevent mortality, morbidity and complications from many infectious diseases in children. It is estimated that around 3 million deaths are prevented each year worldwide through vaccination, and it allows 750 000 children each year avoid suffering from serious physical, mental or neurological disabilities.^1^ In 1974, the World Health Organization (WHO) launched the Expanded Program on Immunisation (EPI) to make vaccines available to all children around the world.^2^ The introduction of the WHO EPI through combined routine and mass immunisation programs against the six deadly childhood diseases (tuberculosis, diphtheria, tetanus, pertussis, polio and measles) has significantly contributed to reducing infant mortality and morbidity.^3^

Despite considerable progress in achieving high immunisation coverage, one in five children in Africa does not receive these essential vaccines.^4^ To address this situation, WHO adopted in 2012 the Global Immunisation Action Plan (2011–2020).^5^ This plan required all countries to achieve immunisation coverage of more than 90% of all antigens by 2020. Also, the introduction of new vaccines was recommended to national immunisation programs.^6,7^

Few countries in Africa have achieved this goal. For instance, only 27 countries have succeeded in introducing the pneumococcal conjugate vaccines (PCV), and the Rotavirus vaccine has been induced by 11 countries.^8^ This demonstrates the challenge that African countries face in achieving the objectives of the plan. Despite this, progress has been made by some immunisation programs. In addition, immunisation coverage varies from one country to another and from one source to another because of the quality of data in different countries. In Ghana, for example, a community survey vaccination coverage in children aged 12 to 23 found that 89.5% (537/600) of them were completely immunised, 9.5% partially immunised and 1.0% received no vaccine.^9^ Another survey of health providers and their children in Nigeria reported an estimated immunisation coverage of 84.9%.^10^ In Senegal, according to data from the Demographic and Health Survey (DHS), the coverage of fully immunised children increased to 62.8% in 2011 to 77.0 % in 2019^11,12^ with disparities between regions.^13^

In Guinea, the EPI recommends that infants be vaccinated with the following vaccines: one dose of Bacillus Calmette-Guerin (BCG) vaccine at birth; three doses of pentavalent vaccine at 6, 10 and 14 weeks; at least three doses of oral polio vaccine (OPV) administered at birth 6, 10 and 14 weeks of age and a dose of measles and yellow fever vaccine at 9 months of age^14^ Despite the recommendations of the WHO, the country has yet to successfully introduce pneumococcus (PCV) and rotavirus vaccines.^8^

The main EPI donors in Guinea are the Global Alliance for Vaccines and Immunisation (GAVI) and other international partners including WHO and United Nations of International Children’s Emergency Fund (UNICEF).^15^ These partners provide technical and financial support to help maintain immunisation coverage at its best level. Furthermore, between 2000 and 2019, the program received $66 862 618 (USD) in assistance from the GAVI Alliance to support the program even after Ebola.^15^ However, despite this high level of funding, the country is struggling to achieve adequate immunisation coverage.

From 2012 to 2018, vaccination coverage fell by 35% (from 37% to 24%) according to DHS data with an increase (from 11% to 22%) in the proportion of children who had not received any dose of vaccine since birth.^14^

Determinants of immunisation coverage in children have been little studied in Guinea. To our knowledge, the available evidence was published in 1990 and 1991, where the socio-demographic factors of the mother were associated with a decline in vaccination coverage of children.^16,17^ Health structures, mainly community health facilities, constitute the first primary point of care for children to access the country’s health system.

In this study, we aimed to estimate the vaccination coverage of children aged 0 to 59 months attending a health centre and identify factors that were associated with incomplete vaccination coverage.

## Methods

### Study site and population

The study was carried out at the Saint Gabriel dispensary of Matoto in the Guinea’s capital Conakry. The dispensary was established in 1987 and provides maternity services or prenatal consultation, vaccination services for children and adults, emergency, nutritional supportive care, laboratory and pharmaceutically services to an estimated population of 48 651 inhabitants, amongst which approximately 13 625 (2.8%) children were under 1 year of age.

In this study, we included all children aged 0 to 59 months who attended primary curative consultations or at the EPI service inform 13 January to 15 February 2020 at the Saint Gabriel dispensary and whose parents gave an informed consent to participate. The children were included according to a systematic random sampling strategy.

Children aged 0 to 59 months who were admitted to emergency rooms, hospitalised or awaiting referral, as well as those accompanying their parents for reasons other than a health visit, were not accounted for in this survey.

### Data collection

The data were collected by a trained interviewer using a pre-tested standardised questionnaire.

Two sources of information include the vaccination card shown by mothers to interviewers and the mother’s recall of vaccination. If the health card was available, information regarding the date of administration was directly collected from the vaccination card which normally records dates of all routine vaccinations. If no card was presented, the interviewer would ask the mother to recall all vaccination received by their child and when appropriate, the number of doses received without asking for the dates. We collected the socio-demographic characteristics of the child (age, sex, birth order, place of birth, reason for consultation (vaccine appointment or disease) and those of the mother (age, profession, marital status, number of prenatal consultations).

*Fully immunised* was defined as a 12 to 23-month-old child who has received BCG, at least three doses of pentavalent vaccine, three doses of OPV and a dose of measles vaccine and yellow fever vaccine.

*Up-to-date* with the immunisation schedule *was* a child (0 to 11 months) who had received all age appropriate vaccines.

*Unvaccinated* – was defined as a child who did not receive any doses of the nine vaccines included in the Guinea EPI schedule.

### Data analysis

Data were collected using Kobo collect software, exported to Excel for cleaning and analysed using Stata version 14. In the descriptive analysis, we calculated frequencies and constructed cross-tabulations for qualitative variables; medians of quantitative variables, as appropriate were computed and reported.

Factors associated with incomplete vaccination coverage were analysed using univariate and multivariate logistic regression. Variables with *p* ≤ 0.20 in univariate analysis were used in multivariate models. Accuracy of the multivariate model was assessed using the Hosmer-Lemeshow test and we used the receiver operating characteristic (ROC) curve to assess the discriminating power of our models. Two multivariate logistic regression models were constructed: One for children aged 0 to 11 months and one for children 12–59 month (see Appendix 1).

### Ethical considerations

The study was approved by the research committee of the public health department of Gamal Abdel Nasser University in Conakry. This article followed all ethical standards for research without direct contact with human or animal subjects.

## Results

### Socio-demographic characteristics of children and their mothers

During the study period, data from 380 children aged 0 to 59 months including 218 children aged 0 to 11 months (57.4%) and 162 children aged 12 to 59 months old were collected. In our study, 248 (65%) children had a health record. The median age of children was 9 months (interquartile range [IQR]: 3 to 20 months). Information on vaccinations status was collected the vaccination records (65.3%) and maternal report (34.7%). The median age of the mothers was 26 years (IQR: 23 to 30 years). The majority (353/380; 92.9%) of the mothers were married and 46.1% had more four prenatal consultation. Most of the mothers (83.4%) were not aware of the next vaccination schedule and the EPI target diseases (Table 1).

**TABLE 1:** Characteristics of study participants and their mothers.

Variables	(*N* = 380)	%
**Children**		
**Age**		
0 to 11 months	218	57.4
12 to 59 months	162	42.6
**Sex**		
Male	200	52.6
Female	180	47.4
**Place of birth**		
Health structure	342	90.0
Home	38	10.0
**Birth order**		
First	89	23.4
Second	115	30.9
Third and more	176	46.3
**Residence**		
Matoto	234	61.6
Other towns in Conakry	80	21.1
(Matam, Ratoma, Dixinn) Coyah or Dubréka	66	17.4
**Reason for consultation**		
Vaccination appointment	130	34.2
Disease	250	65.8
**Sick child last month**		
Sick child	172	45.3
Child not sick	280	54.7
**Mothers of children Age (range)**		
Under 25	205	53.9
25 years and over	175	46.1
**Marital status**		
Married	353	92.9
Divorcee	3	0.8
Single	22	5.8
Widow	2	0.5
**Religion**		
Muslim	343	90.3
Christian	37	9.7
**Mother’s activity**		
With source of income	192	50.6
Without source of income	188	49.4
**Prenatal consultation performed during pregnancy**		
One to two prenatal consultation	138	36.3
Three prenatal consultation	67	17.6
Four or more prenatal consultation	175	46.1
**Information on diseases to be avoided by vaccination**		
Yes	63	16.6
No	317	83.4
**Information on side effects of vaccines**		
Yes	211	55.5
No	169	44.5

### Vaccination coverage of children surveyed

One-hundred forty-five (66.5%; 95% confidence interval [CI]: 59.8–72.7) of 218 infants aged 0 to 11 months were up-to-date with their immunisations. Two-hundred ten (96%; 95% CI: 92.9–98.4) infants of 218 or 96% 95% CI (92.9–98.4) had received the first doses of BCG and OPV vaccines at birth. Six (2.8%) of the children in this age group had not received a vaccine (Figure 1).

**FIGURE 1:**
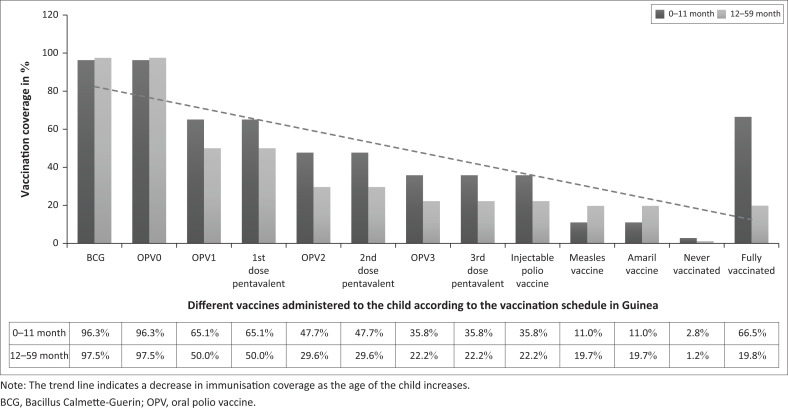
Attrition in vaccination coverage over time amongst study participants.

In the 162 children aged 12 to 59 months, vaccination coverage was complete in 19.8% 95% CI (13.9–26.7) and two (1.2%) of them had not received any vaccine. Most 97.5%; 95% CI (93.8–99.3) of these children receive birth doses of BCG and OPV vaccines, 50% (95% CI: 42.0–57.9) received the first dose of pentavalent vaccine and OPV1 and 22% (95% CI: 16.1–29.4) received the third dose of pentavalent vaccine and OPV3.

Factors affecting vaccination coverage in univariate analyses are shown in Table 2. Statistically, significant associations were noted between lack of vaccination coverage and the child’s birth order (*p* < 0.001), history of illness in the month prior to scheduled vaccination (*p* < 0.001) and lack of knowledge about vaccine-preventable diseases (*p* < 0.001). Boys were vaccinated significantly more frequently than girls were (*p* = 0.01). Residence in Matoto was associated with better vaccination status (*p* = 0.03).

**TABLE 2:** Characteristics of study participants stratified by vaccination status.

Variables	Immunisation schedule status				*P*
	Up to date		Not up to date		
	*n*	%	*n*	%
**Have a vaccination card**					< 0.001
Yes	150	85.7	98	47.8	
No	25	14.3	107	52.2	
**Sex**					0.010
Male	104	59.4	96	46.8	
Female	71	40.6	109	53.2	
**Place of birth**					0.050
Health structure	163	93.1	179	87.3	
Home	12	6.9	26	12.7	
**Child’s birth order**					< 0.001
First	61	34.9	28	13.7	
Second	72	41.1	43	13.7	
Third or higher	42	24.0	134	65.4	
**Residence**					0.030
Matoto	120	68.6	114	55.6	
Other towns	30	17.1	50	24.4	
Coyah/Dubréka	25	14.3	41	20.0	
**Antecedents disease months before vaccination**					< 0.001
Sick child	26	14.9	146	71.2	
Child not sick	149	85.1	59	28.8	
**Reasons for consultation**					< 0.001
Vaccination appointment	113 5	65.6	17	8.3	
Disease	62	35.4	188	91.7	
**Mother’s age (range)**					0.730
Under 25	85	48.6	96	46.8	
Over 25 years	90	51.4	109	53.2	
**Marital status**					0.230
Married	167	95.4	186	90.7	
Divorcee	1	0.6	2	1.0	
Single	7	4.0	15	7.3	
Widow	0	-	2	1.0	
**Religion**					0.870
Muslim	153	87.4	190	92.7	
Christian	22	12.6	15	7.3	
**Mother’s occupations**					0.070
Mother’s activity	105	60.0	141	68.8	
With source of income	70	40.0	64	31.2	
**Number of prenatal consultations performed**					< 0.001
Four or more prenatal consultation	114	65.1	61	29.8	
Three prenatal consultation	35	20.0	32	15.6	
One to two prenatal consultation	26	14.9	112	54.6	
**Disease Information to be avoided by vaccination**					< 0.001
Yes	58	33.1	5	2.4	
No	117	66.9	200	97.6	

### Factors associated with incomplete immunisation coverage in children

Two factors were associated with incomplete immunisation coverage in children aged 0 to 11 months (Table 3, Appendix 1). These were unavailability of vaccination records (adjusted odds ratio [aOR] = 7.58; 95% CI: 2.56–22.44) and lack of prenatal consultation attendance (aOR = 2.93; 95% CI: 1.15–7.48). If a child was well in the preceding month, vaccination coverage was, on an average, 88% (95% CI: 71–95) better (Table 4).

**TABLE 3:** Factors associated with incomplete vaccination coverage in children aged 0 to 11 months, Guinea, January–February 2020.

Variables	Univariate regression			*P*	Multivariate regression			*P*
	Odds ratio	95%	Confidence interval		Odds ratio	95%	Confidence interval	
**Sex**								
Male	1	-	-	-	1	-	-	-
Female	-	1.30	0.74–2.30	0.350	-	1.27	0.60–2.69	0.510
**Child’s place of birth**								
Health structure	1	-	-	-	1	-	-	-
Home	-	1.17	0.44–3.12	0.740	-	0.90	0.22–3.61	0.890
**Birth order**								
First	1	-	-	-	1	-	-	-
Second	-	0.90	0.43–1.87	0.780	-	1.23	0.48–3.19	0.650
Third and more	-	2.55	1.25–5. 21	0.010	-	1.58	0.44–5.67	0.480
**Availability of a vaccination record**								
Yes	1	-	-	-	1	-	-	-
No	-	10.90	4.44–26.76	< 0.001	-	7.58	2.56–22.44	< 0.001
**Child’s state of health last month**								
Sick child	1	-	-	-	1	-	-	-
Child not sick	-	0.10	0.05–0.20	< 0.001	-	0.12	0.05–0.29	< 0.001
**Mother’s age (range)**								
Under 25	1	-	-	-	1	-	-	-
25 years and over	-	0.90	0.51–1.60	0.740	-	0.45	0.16–1.22	0.110
**Mother’s activity**								
With source of income	1	-	-	-	1	-	-	-
Without source of income	-	1.97	1.11–3.49	0.020	-	1.15	0.51–2.55	0.720
**Numbers of antenal consulting performed during pregnancy**								
Four prenatal consultation or more	1	-	-	-	1	-	-	-
Three prenatal consultation	-	1.71	0.78–3.74	0.170	-	1.27	0.50–3.21	0.600
One to two prenatal consultation	-	6.50	3.23–13.09	< 0.001	-	2.93	1.15–7.48	0.020

**TABLE 4:** Factors associated with incomplete vaccination coverage in children aged 12 to 59 months in Matoto, Guinea January to February 2020.

Variables	Univariate regression			*P*	Multivariate regression			*P*
	Odds ratio	95%	Confidence interval		Odds ratio	95%	Confidence interval	
**Sex**								
Male	1	-	-	-	1	-	-	-
Female	-	1.45	0.66–3.15	0.340	-	2.38	0.80–7.03	0.110
**Child’s place of birth**								
Health structure	1	-	-	-	1	-	-	-
Home	-	4.98	0.64–38.80	0.120	-	2.47	0.19–22.11	0.480
**Child’s birth order**								
First	1	-	-	-	1	-	-	-
Second	-	2.13	0.69–6.62	0.180	-	1.78	0.37–8.59	0.470
Third and more	-	13.58	4.44–20.37	< 0.001	-	10.29	2.06–19.43	< 0.001
**Availability of a vaccination record**								
Yes	1	-	-	-	1	-	-	-
No	-	1.28	0.58–2.81	0.520	-	1.23	0.41–3.68	0.700
**Has the child been sick in the last month?**								
Yes	1	-	-	-	1	-	-	-
No	-	0.58	0.02–0.16	< 0.001	-	0.17	0.05–0.54	0.003
**Mother’s age**								
Under 25	1	-	-	-	1	-	-	-
25 years and over	-	0.75	0.34–1.64	0.470	-	0.253	0.17–2.29	0.480
**Does the mother have a source revenue**								
Yes	1	-	-	-	1	-	-	-
No	-	3.48	1.49–8.11	0.004	-	1.78	0.33–3.89	< 0.310
**Numbers of antennal consulting performed during pregnancy**								
Four or more prenatal consultation	1	-	-	-	1	-	-	-
Three prenatal consultation	-	6.15	1.31–28.83	0.021	-	3.13	0.49–19.66	0.220
One to two prenatal consultation	-	12.65	4.11–38.91	< 0.001	-	5.34	1.48–19.23	0.010

Factors associated with incomplete immunisation coverage in children 12 to 59 months of age were third birth order aOR = 10.29; (95% CI: 2.06–19.43), lack of prenatal consultation attendance (aOR = 5.34; 95% CI: 1.48–19.23). If the child was well in the preceding month, vaccination coverage was, on an average, 86% (95% CI: 51–96) better (Table 4).

## Discussion

Of the 380 children aged 0 to 59 months included in this study, 65.3% had a vaccination record, whereas 34.7% had their vaccination status determined through maternal report of those with available vaccination records and 80% 5304/380) were less than 1 year old. Absence of a vaccination record was strongly associated with incomplete vaccination in this study. This factor has been found to be a predictor of non-compliance with the vaccination schedule in various studies. The results of a study carried out in Senegal^12^ showed that children whose mothers presented a vaccination card, and who attended at least a secondary level of education were more likely to be fully immunised. Similarly, Adokiya et al.^9^ in Ghana showed that children whose immunisation cards were not available at the time of the study were more likely not to be fully immunised than children whose immunisation cards were available.

Another study performed in Cameroon yielded the same results.^18^ The use of electronic children’s health records could alleviate this problem.

In our study, male children had higher immunisation coverage than female children (*p* = 0.01). In African culture, boys are often considered as heirs and therefore enjoy a lot of attention which may result in more accessibility to care. A study from Bangladesh found that boys received more food than girls.^19^

A high birth order was associated with poor vaccination coverage in our study, in which 46.3% of the children were the third child or greater. This may reflect family or maternal focus on immunising their first- or second-born children but failure to ensure adequate immunisation coverage in subsequent children, possibly as a result of complacency, or fall-out because of fatigue in attending routine clinic appointments. Birth order has been associated with childhood immunisation incompleteness in other studies.^16^

Childhood immunisation coverage of mothers over 25 was better than children of younger mothers. Mohamed et al. in Ethiopia^20^ made the same observation in their study. Older mothers are more experienced and mature, so they pay more attention to their children.

Prenatal consultations are an ideal time not only to prepare for childbirth but also to create the conditions for better health of the future child. The WHO recommends that women have at least four or more prenatal consultations during pregnancy. In our study, less than half (46.1%) of mothers attend four or more prenatal consultation appointments. prenatal has been described in several studies.^20,21^ Immunisation coverage of children of mothers who attended four or more prenatal consultation was 65.1% versus 14.9% of children whose mothers attended fewer less than two prenatal consultation (*p* < 0.001). Low utilisation of health services and missed opportunities for delivery of health education at prenatal consultation would be expected to decrease the chances of optimising care of the infant delivery.

In this study, 145 (66.5%) infants aged 0 to 11 months had received all vaccines according to the vaccination schedule at the time of the survey. This coverage is lower than that found in South Africa (73%),^22^ and Cameroon (88%),^23^ but similar to rates seen in the US Medicaid Program in the 1980s (50%).^24^ These differences could to some extent be explained by different study designs.

Lack of immunisation in infancy is concerning, as infants, who have relatively reduced immunity, are at risk of diseases targeted by immunisation programs.

Delays in accessing vaccination services may be because of concerns about post-vaccination side effects in some children, neglect or forgetting the date of the EPI appointments.^25^

Only, 19.8% (32/162) of children aged 12 to 59 months were fully immunised in our study.

This result corroborates the findings of the Guinea Demographic and Health Survey 2018 (DHS) in which 24% of children were completely vaccinated,^14^ and is inferior to data from the study by Deressa et al.^26^ in Ethiopia who found that 35.4% of children were completely vaccinated.^14^ There is a disconnect between administrative and WHO/UNICEF estimates of vaccination coverage in Guinea, in which DTP3 vaccination coverage in 2018 was estimated at 75% and 45%, respectively.^27^ There has been no improvement in immunisation.

Coverage for children aged 12 to 23 months between the two previous DHS in Guinea and administrative coverage is still higher than the results of one of surveys because of the lack of updated data on the target population and calculation of immunisation coverage based on the number of vaccine doses used.

Immunisation coverage was inversely proportional to the child’s age in our study. Most children (98%) received a birth dose of BCG and OPV, 50% received the first dose of pentavalent vaccine and OPV1 and 22% received the third dose of pentavalent vaccine and OPV3. High rates of omission of the last doses of pentavalent vaccine and doses of measles and yellow fever vaccines were concerning.

Higher rates of immunisation coverage (BCG-OVP0 99%, first dose of pentavalent vaccine-OVP1 98%, third dose of pentavalent vaccine-OVP3 95%) were noted by Saker et al.^19^ in Senegal. The large attrition rate between the 14-week vaccinations (third dose of pentavalent vaccine and OPV3) and receipt of yellow fever and measles vaccine (scheduled for 9 months of age) could be linked to the 6 month scheduled vaccination appointments. Establishment of telephone call-back programs for mothers, reminding them to attend scheduled vaccination visits with their infants, may be a solution to this problem.

We assessed maternal knowledge of EPI and vaccine preventable diseases in large proportion (83%) and did not know which diseases children should be immunised against. This finding is lower than that of Etana and Deressa^26^ in Ethiopia who showed that 96% of mothers had heard of childhood immunisations and vaccine-preventable diseases. In our study, 55.5% of mothers were aware of the side effects likely to occur in some children after vaccination, lower estimates from Senegal where 87.9% of mothers were aware of at least one post-injection manifestation.^13^ The explanation may be linked to maternal educational status but may be exacerbated through lack of information on hazards and vaccine preventable diseases in local languages through radios and television shows.

## Conclusion

Immunisation coverage of children under five remains low in Guinea, and coverage decreases with age whith. children more likely to receive vaccines at birth than 9 months of age. Mothers are not made aware of the vaccination schedule and appear to receive little information on adverse events following immunisation. Factors that were significantly associated with low immunisation coverage in our study were included. Incomplete vaccination record, low attendance of prenatal care by mother, increasing, birth order, missing child health records and lack of income. It is, therefore, important that hospital health workers educate mothers about the childhood immunisation program and potential side effects of vaccines.

Moreover, the government should aim at increasing the use of health services during pregnancy and postpartum.

## Figures and Tables

**FIGURE 1:**
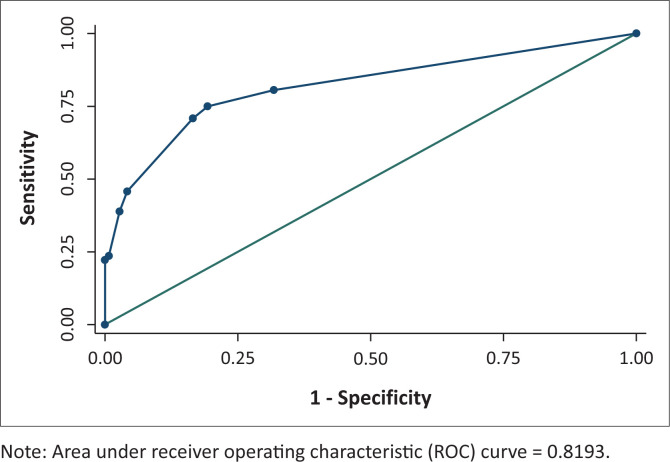
Area under ROC curve, logistic regression for factors associated with incomplete vaccination coverage in children aged 0 to 11 months.

**FIGURE 2:**
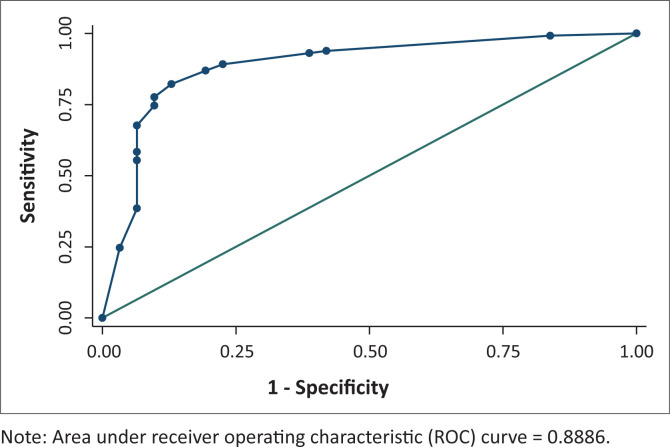
Area under ROC curve logistic regression for factors associated with incomplete vaccination coverage in children aged 0 to 12 months.
